# Health inequalities research in India: a review of trends and themes in the literature since the 1990s

**DOI:** 10.1186/s12939-016-0457-y

**Published:** 2016-10-06

**Authors:** Nandita Bhan, Krishna Dipankar Rao, Shivani Kachwaha

**Affiliations:** 1 Public Health Foundation of India, New Delhi, India; 2 Johns Hopkins University, Baltimore, USA; 3 The Graduate Institute of International and Development Studies Geneva, Geneva, Switzerland

**Keywords:** Health inequalities, Equity, Socioeconomic status, Social gradient, India

## Abstract

**Background:**

Research on health inequalities can be instrumental in drawing attention to the health of socioeconomically vulnerable groups in India in the context of rapid economic growth. It can shape the dialogue for public health action, emphasizing the need for greater investments in health, and monitor effectiveness of health programs. Our objective was to examine trends in studies on health inequalities in the last 25 years.

**Methods:**

We conducted a systematic literature review of studies on health inequalities published from 1990. The year, 1990, marked the beginning of economic reforms and liberalization in India. We searched PubMED using key terms to identify 8800 articles between 1990 and 2016; we identified 1,312 final studies for review. Key domains of analysis included measures of equity, health outcomes, populations studied, year of publication, study methodology, study focus (descriptive versus analytical), and location of main author.

**Results:**

We found an increase in studies on health inequalities after 2005. About 88 % of the studies utilized quantitative methods for analysis. About 8 % of the studies related to health interventions or programs; the number of intervention studies have been increasing since 2010. A majority of studies were led by authors based in India. Early studies focused on mortality, communicable and non-communicable diseases, and nutrition, while later studies have focused on non-communicable diseases, mental health, risk factors, and injuries. Studies on women and children comprised nearly half of the literature; studies on the youth (15–24 years or as defined by the study) and elderly have been rising. Wealth and income were the most common measures of equity, followed by education and gender. The proportion of studies on wealth, education, region and caste have stayed consistent over time, while studies on gender disparities have been rising.

**Conclusion:**

In a country as diverse as India with large social inequalities combined with rapid economic growth, research on health inequalities has a special significance for policy. We recommend that studies on health inequalities in the future focus on evaluations of policy and health programs, and on underrepresented health outcomes and populations.

## Background

Research on health inequalities has been instrumental in drawing attention to the health of socially and economically vulnerable groups in India. It has shaped the dialogue for public health action, emphasized the need for greater and targeted investments in health, and can be an important marker for the effectiveness of public health services [1–5]. Importantly, health inequalities research enriches our understanding of societal disparities in health and healthcare, moving away from a narrow focus on income to include markers of deprivation like gender, caste, religion and occupation that afflict health and the quality of life. Research on health inequalities has now become one of the central pillars of the development dialogue [6–9]. This research profoundly influences health policy; the recent call for universal health coverage (UHC) represents the latest effort to reduce health disparities globally and in India [8, 10–12].

Prior to the 1980s, few studies investigated health inequalities. There may be several reasons for this. Post-independence, the focus of health policy was increasing the coverage of health services and hence, research focused on the delivery of basic health services. Philosophical debates on nation-building and the role of modern medicine also led to a shift from investigations of the role of caste, religion and ethnicity [13, 14]. Socioeconomic stratifiers were considered ‘shackles’, holding India back from modernization [15, 16]. This vision and the recommendations of the Bhore Committee led to a number of clinical research studies [17]. The Bhore committee was set up in 1943 under Sir Joseph Bhore to make recommendations on improving public health system in India. The report envisioned a national health system that was tax funded and publicly run along the lines of the Beveridge model adopted in the United Kingdom. At the other end, social science studies were mainly investigating micro-perspectives and health practices of specific cultural groups leading to village studies, anthropological assessments and historical analyses [18–24].

In 1964, the Indian Journal for Medical Research (IJMR), a bi-annual journal of the ICMR, was turned to a monthly publication, increasing the scope for publishing more studies. Until then, few research journals published health studies and research and training in public health was the domain of physicians and departments of community medicine within medical colleges [25]. However, most published studies were clinical and the objective was to identify new clinical conditions and issues in different parts of India. Few studies focused on assessing health inequalities. The policy sphere was also devoid of discussion on health inequalities. Different governmental committees were engaged in evaluating health service delivery and improving coverage of health services [26–28].

A series of global and national events brought health inequalities on the policy radar. The Alma Ata ‘Health for All’ declaration emphasized the significance of primary health care for reducing health disparities [29]. People’s movements for health highlighted multiple deprivations faced by vulnerable groups globally. In Brazil, for instance, after 20 years of political dictatorship, movements for health as a fundamental right led to a constitutional amendment in 1988, based on the principle of reducing health disparities through a responsive public health infrastructure [30]. In India, the focus of research in the 1980s was on family planning, reproductive health and child survival [31–34]. Policy emphasis on the role of women in reducing health disparities in reproductive and child health led to mainstreaming of gender in research [35, 36]. Gender and poverty were considered the structural determinants of health inequalities in maternal and child health. Health inequalities research also received a push with the WHO Commission on Social Determinants of Health (CSDH) [37, 38]. This commission emphasized the importance of systematically investigating the role of social inequalities, particularly living conditions, for health.

Social and political movements also played an important role in highlighting inequalities. The landmark report on gender, ‘Towards Equality’, highlighted socioeconomic challenges faced by women in diverse domains of life [39]. Poverty and education have been central to Indian public policy and health [40–50]. Low rates of education among women was considered a major barrier in achieving health goals [2, 31]. Movements related to caste, region and religion have also contributed to improving our understanding of inequalities [51–53].

Developments in health education, particularly the setting up of departments of preventive and social medicine in medical schools and health electives in other courses such as in social work built capacity for research on health inequalities in India [25]. Training and course development in these streams improved capacity for conducting field epidemiological studies. Early studies on the health burden emerged from surveillance sites set up by independent research groups affiliated to universities. The Indian Association of Preventive and Social Medicine (IAPSM) (1974) and launch of the Indian Journal of Community Medicine (IJCM) enhanced avenues for discussion and publication of research on health issues and the scope of training and research in these institutes.

A paradigm shift for research on health inequalities in India was seen with public availability of survey data. A Ministry for Statistics and Program Implementation had been set up shortly after independence to plan and conduct decennial census, population surveys and surveillance studies. However, for decades only few statistics were available in the public domain and raw data could not be acquired. The National Sample Survey Organization (NSSO) provided the first national level assessments of self-reported health and health care data on a cross-section of social and policy themes. Nutrition surveys conducted by the National Institute of Nutrition (NIN) gathered data on food and nutrition; this data remains underutilized. In the 1990s, the USAID funded the Demographic Health Surveys (DHS), a multi country cross-sectional survey focused on reproductive health and family planning [54]. In India, this was referred to as the National Family Health Survey and data are now available for three rounds (1992–93, 1999–2000 and 2005–06). In the 1980s, the national government also released the National Sample Surveys (NSS) which collect data from households on consumption, labor force participation and other key development themes [55]. Both these surveys led to a flurry of studies on health inequalities as data became available to both international and national researchers [56, 57]. These research studies have added depth and richness to the understanding of health inequalities in India. While research on social inequalities including gender, caste, poverty and education is conducted by social scientists, research on health inequalities has been driven by cross-disciplinary groups of epidemiologists and social science scholars.

The **main objective** of this study was to examine the direction of health inequalities research in India over the last three decades to understand key patterns, themes and trends. With this **research question**, we conducted a review of published peer-reviewed studies on health inequalities in India to understand the issues examined and key gaps in present research.

## Methods

In this study, we reviewed published studies to examine trends in health inequalities research in India since 1990, which marked the onset of economic reforms in India. We describe the populations in focus, main methods utilized, health outcomes studied and measures of equity used in the research on health inequalities in India.

### Search

We conducted a systematic literature search on PubMED, the database of the US National Library of Medicine for research on health inequalities published between 1990 and 2016. PubMED is a widely used online bibliographic database for public health and medicine and indexes a large number of international and national journals. We considered publications from 1990 onwards for two reasons. Firstly, 1990 marked the beginning of economic reforms in in India which has implications for equity in health and healthcare. Additionally, the period from 1990 to 2016 covers a time period of more than 25 years of research publications. Databases were searched using the terms: ‘*India*’, ‘*health status disparities*’, ‘*healthcare disparities*’, ‘*health services needs and demand*’, ‘*social justice*’, ‘*social marginalization*’, ‘*poverty*’, ‘*socioeconomic factor*’, ‘*social class*’, ‘*ethnic groups*’, and ‘*minority health*’. We followed relevant aspects of the PRISMA guidelines for the literature search, for defining key aspects of the study methodology and in drafting the manuscript.

### Study selection

We used search terms to review and identify relevant study abstracts. We included studies that reported single or multiple types of health inequalities, studies on the burden of diseases reporting stratification by socioeconomic factors, studies showing gender differences, multi country studies that reported disparities for India and any other studies of studies that included data on health inequalities. We excluded duplicates, clinical studies, studies on the burden of diseases that did not stratify by any socioeconomic or demographic factors, reviews, editorials or any other studies of articles, studies on Indian populations in other countries and studies showing differences in health outcomes by age groups only.

### Data extraction

Following this, information was extracted from identified abstracts and full text studies. Domains of data extraction included information on year of publication, study methodology, study focus (descriptive versus analytical), location of main author, health outcomes of focus, population group and measures of socioeconomic disparity.

### Summary measures

In particular, we examined population groups, health outcomes and measures of disparity in focus. Extracted data was summarized into spreadsheet templates and results were synthesized quantitatively.

### Data items and synthesis of results

We classified studies into nine thematic sub-groups. These included studies on mortality, communicable diseases, non-communicable diseases, mental health, injuries, health services, risk factors, malnutrition and others. Key population groups considered by the study included men, women, adults (i.e. men and women), child (including adolescents), youth (age group of 15–24 years or as defined by the study), elderly and all populations (for studies that did not specify a population group). We classified measures of socioeconomic disparities into geographical markers (rural, urban or states), income or wealth, occupation, education, religion, caste/tribe, gender and access to water/sanitation. If studies reported multiple socioeconomic markers, all of them were recorded in the analysis.

## Results

**Fig. 1:**
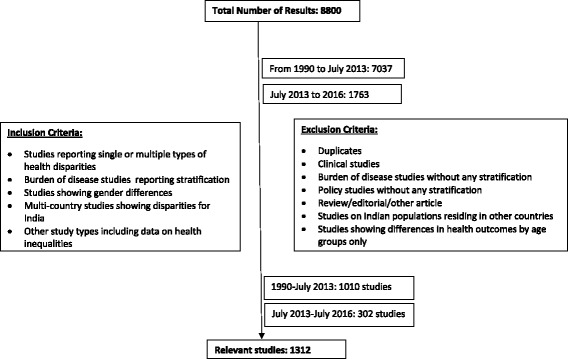
Review of Literature on Health Inequalities

A review of research published on health inequalities between 1990 and 2016 yielded 8,800 abstracts of which 1,312 studies were relevant to this review (Fig. 1).

### Publication trends over time

**Fig. 2:**
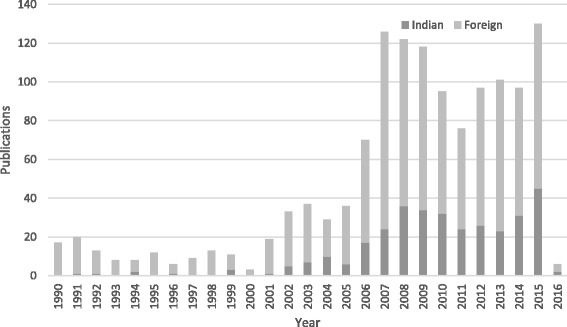
Published studies on health inequalities between 1990 and 2016 by country of lead author. *Note: Data for the year 2016 is till July only

We found an increase in the research published on health inequalities, especially after 2005 (Fig. 2). Our review shows that of the studies published, more than 9 % were published between 1990 and 2000, and 90 % were published between 2001 and 2016. Among the studies published, a majority (75 %) were led by authors based in Indian institutions. The share of lead authors based at international institutions was small but growing (Fig. 2). Studies led by international researchers originated in developed nations such as the United States of America (USA), United Kingdom (UK), European Union (EU) countries (Germany, France, Denmark, Sweden and Ireland) and Australia.

### Research methods

More than 88 % of the studies identified by the review used quantitative methods, with 4.1 % using qualitative methods and 4.5 % using mixed methods. Over time, we noted a decline in the proportion of qualitative and mixed methods studies from 6.7 and 5.8 % respectively in the 1990s to 3.8 and 4.4 % respectively between 2000 and 2016. The proportion of quantitative studies increased from 84 % in the 1990s to 89 % between 2000 and 2016. Nearly 92 % of the studies were descriptive or comparative in nature, with 7.9 % investigating the impact of health interventions and programs. Since 2010, intervention studies are on the rise.

### Health outcomes

**Fig. 3:**
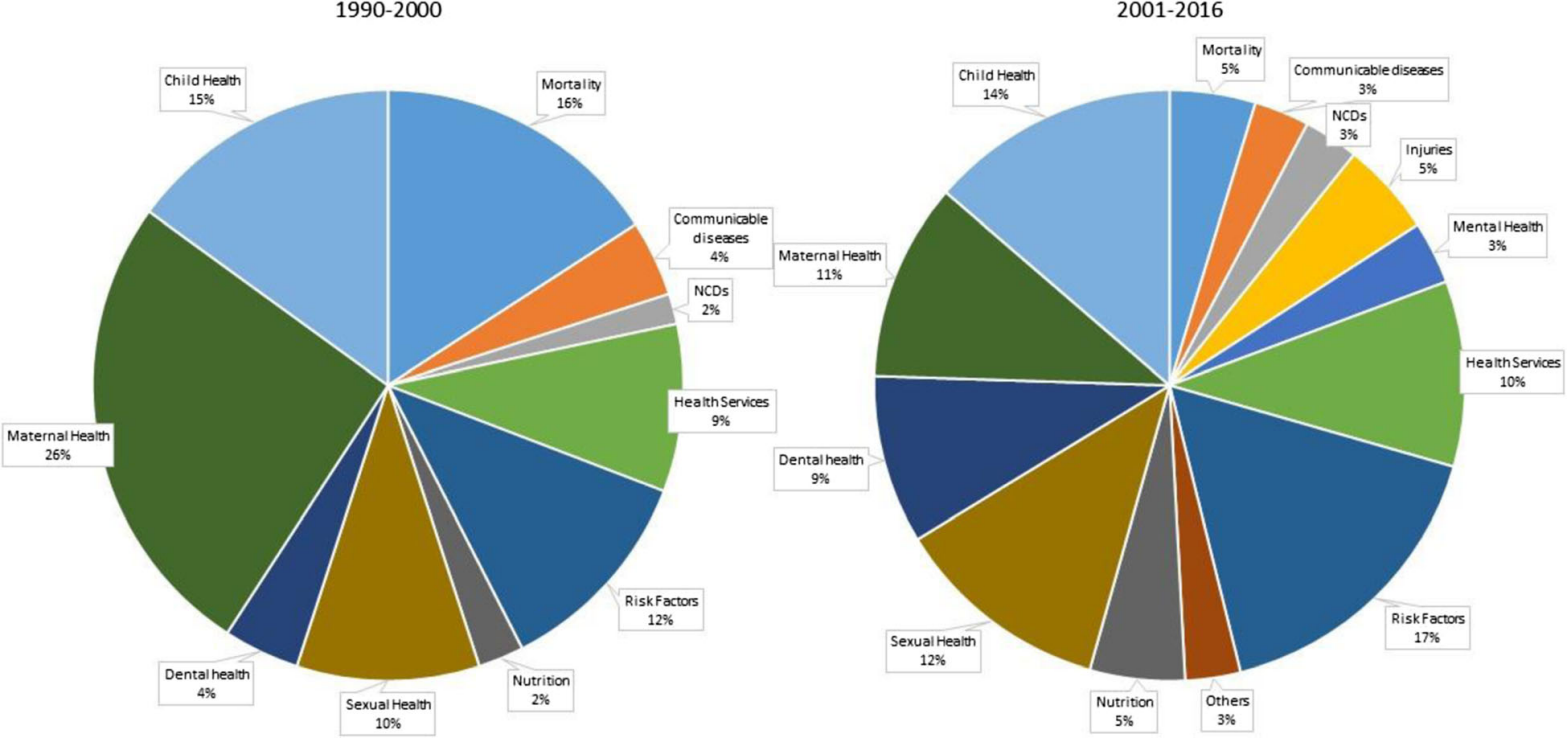
Distribution of Study Outcomes in the Health inequalities Research (1990–2016)

Communicable and non-communicable diseases and malnutrition comprised the largest proportion of the research literature (Fig. 3). Of the studies reviewed, 10.1 % examined disparities in communicable diseases, 16.2 % in non-communicable diseases and 13.8 % focused on nutrition. Studies on health services comprised 11.7 % of the total studies reviewed and the category ‘Other’ comprised 12.2 %. Studies on risk factors comprised 8.8 % of the reviewed studies. About 5.7 % of the studies focused on mortality, 4.5 % on mental health and 4.6 % on maternal health.

Studies prior to 2000 focused on mortality, communicable and non-communicable diseases. Studies on mortality declined after 2000 and studies on non-communicable diseases started increasing between 2000 and 2016. We did not find any studies on injuries or accidents prior to 2000, but between 2000 and 2016, 3 % of the studies examined injuries. Studies on mental health doubled from 2.5 % (1990–2000) to 5.2 % (2000–2016), while the proportion of studies on health services declined. The number of studies on risk factors and nutrition increased between 2000 and 2016. Research on sexual health and dental health emerged as defined areas from 2000 onwards comprising 3 % each of the total literature respectively.

### Population groups

**Fig. 4:**
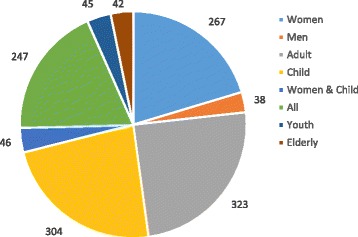
Distribution of Population Groups in the Health inequalities research (1990–2016)

Nearly half of the published studies focused on women and children (Fig. 4). About 20.3 % of the studies focused exclusively on women, 24.6 % focused exclusively on children and 3.5 % examined outcomes for both women and children. Studies on men comprised only 2.9 % of the total studies and youth and elderly comprised 3.4 % and 3.2 % of the literature respectively.

We noted three trends in the population groups studied by the research literature. Firstly, we found a decline in the proportion of studies on children from 41.7 % (1990–2000) to 21.3 % (2010–2016). Secondly, we found that the proportion of studies focused on all populations increased from 5.1 % in 1990–2000 to 20.2 % in 2000–2016. This may be attributed to diversification of study themes that were earlier focused on maternal and child health. Finally, we noted an increase in studies on youth and elderly. Studies on elderly increased from none in the 1990s to 3.5 % in 2000–16. Studies on the youth populations increased from none in 1990s to 3.8 % in 2010–16.

### Measures of equity

Income (including wealth) (61.2 %) was the most common measure of equity for health disparities, followed by education (45.4 %) and gender (36.1 %) (Table 1). A substantial number of studies considered disparities based on region, occupation and caste/tribe status. The importance of wealth or income stayed consistent over time. The proportion of studies on education, income, region, caste and religion also stayed consistent over time. Studies on gender disparities in health increased over time from 33.3 % (1990–2000) to 38.1 % (2011 onwards).

**Table 1:** Trends in Equity Measures Used in Health Inequalities Research in India (1990–2016)

Disparity measure	Total (%)	1990–2000 (%)	2000–10 (%)	2010–2016 (%)
Region	34.8	25.8	28.6	45.2
Income	61.2	48.3	55.3	72.2
Occupation	15.2	14.2	15.0	15.8
Caste/Tribe	12.9	15.8	11.9	13.6
Religion	8.8	10.8	7.9	9.5
Gender	36.1	33.3	35.2	38.1
Education	45.4	46.7	39.7	52.9

Note: Multiple measures of equity were present in many studies

## Discussion

In a country as diverse as India with large social inequalities combined with rapid economic growth, research on health inequalities has a special significance for policy. The rapidly growing literature on health inequalities further attests that. While health inequalities as a research domain emerged from disciplines like demography, economics and sociology, today it represents a large and interdisciplinary field of study in health research. Studies that show socioeconomic gradients have firmly established health inequalities in the development discourses both internationally and in India [46, 47, 56–60]. However, to an extent research on health inequalities in India follows international trends with local flavor.

We found that despite their gaps, research on health inequalities has systematically highlighted the large disparities across health outcomes that exist in India [56, 57, 61–65]. Outcomes have extended beyond documented differences in health outcomes across groups, to research on the distribution of public subsidies and out of pocket payments for health and impoverishment across socioeconomic groups [61–65]. These studies have been widely used to inform health policy and programs. We also found that studies on interventions have also contributed to measuring how successful health programs have been in reaching disadvantaged groups (and reducing health disparities) [46]. Hence, health inequalities research has also moved beyond documentation to become an important policy tool.

Our review of the literature on health inequalities in the last 25 years provides insight into how the field of research has evolved in India. The number of studies has grown over time and this research has been led by researchers based in India. A majority of studies conducted are quantitative, which shows the important role of quantitative disciplines like epidemiology and economics in this research. The contribution of qualitative studies at present remains largely untapped. We found that a majority of studies were situational analyses with few studies on health programs and interventions. Even as the latter have played an important role for policy, their potential for health inequalities research in evaluation of programs and policies remains underutilized.

The themes investigated in the literature on health inequalities also indicate the changing politics of health issues in public health. We found that studies in the 1990s focused on mortality, communicable and non-communicable diseases and nutrition. However, in the latter period, non-communicable diseases, health risk factors, mental health and injuries acquired increasing importance. These patterns broadly reflect shifts in public health priorities, globally and in India. Studies on women and children have dominated this research in India, attributed to policy focus on reproductive and child health in public health programs. Wealth has been the main socioeconomic marker studied followed by education and gender. This resonates largely with global practice. In the Indian context, caste/tribe status and religion occupy an important position as they capture sociocultural aspects of disadvantage. However, in health inequalities studies, their proportion remains low.

## Conclusion

In a country as diverse as India with large social inequalities, research on health inequalities has a special significance for monitoring effectiveness of health policies and programs. We recommend that future research in this area focuses on evaluations of policies and health programs in order to ensure improved targeting towards underserved populations. We also recommend that future research focuses on underrepresented health areas and populations.

## Abbreviations

CSDH: Commission on Social Determinants of Health
DHS: Demographic and Health Surveys
GATS: Global Adult Tobacco Survey
LMIC: Low and middle income countries
MICS: Multiple Indicator Cluster Survey
NCD: Non communicable diseases
UHC: Universal Health Coverage
